# *Caenorhabditis* Intervention Testing Program: all-trans retinoic acid lifespan extension in *Caenorhabditis elegans* requires 5-Fluoro-2'-deoxyuridine (FUdR)

**DOI:** 10.17912/micropub.biology.002343

**Published:** 2026-08-19

**Authors:** Stephen A. Banse, Anna L. Coleman-Hulbert, Christine A. Sedore, Erik Johnson, Gordon J. Lithgow, Monica Driscoll, Patrick C. Phillips

**Affiliations:** 1 Institute of Ecology and Evolution, University of Oregon, Eugene, Oregon, USA; 2 The Buck Institute for Research on Aging, Novato, California, USA; 3 Department of Molecular Biology and Biochemistry, Rutgers University, Piscataway, New Jersey, USA

## Abstract

The *Caenorhabditis* Intervention Testing Program recently characterized the longevity-promoting effects of all-trans-retinoic acid (atRA) in *C. elegans* . While we observed that atRA extended lifespan across multiple genetic backgrounds, environmental factors may influence its efficacy. One such factor is 5-Fluoro-2'-deoxyuridine (FUdR), used to prevent progeny contamination in lifespan assays. Here, we show that FUdR enhances atRA's lifespan effects, but that this potentiation is not simply due to progeny elimination, as sterilization through auxin-mediated disruption of spermatogenesis does not potentiate atRA in lifespan extension. We conclude that atRA longevity is dependent on additional effects of FUdR that remain uncharacterized.

**Figure 1. atRA longevity effects require FUdR:** Kaplan Meier curves for animals grown under adult exposure to 150 µM atRA (green) or vehicle control (black). All experiments were performed in the *C. elegans* N2 background. Shown are the combined results from two replicates that were all completed at the Oregon CITP testing site. (A) Manual lifespan assay using the auxin tagged *spe-44::aid* strain PX627 (dashed lines) under standard conditions (no auxin, 51 µM FUdR), compared to N2 _PD1073 (solid lines). (B) Manual lifespan assay using the auxin tagged *spe-44::aid* strain (Kasimatis et al. 2018) treated with 1 mM auxin from hatching through day one of adulthood to eliminate self-fertility. The experiment was done in the presence (solid lines) or absence (dashed lines) of 51 µM FUdR. (C) Manual lifespan assay of N2 _PD1073 in the absence of FUdR. All statistical comparisons were made with a Cox proportional hazards (CPH) mixed-model using the coxme v.2.2-22 package in R where asterisks represent p-values from the CPH model such that **** *p* <.0001, *** *p* <.001, ** *p* <.01, and * *p* <.05.

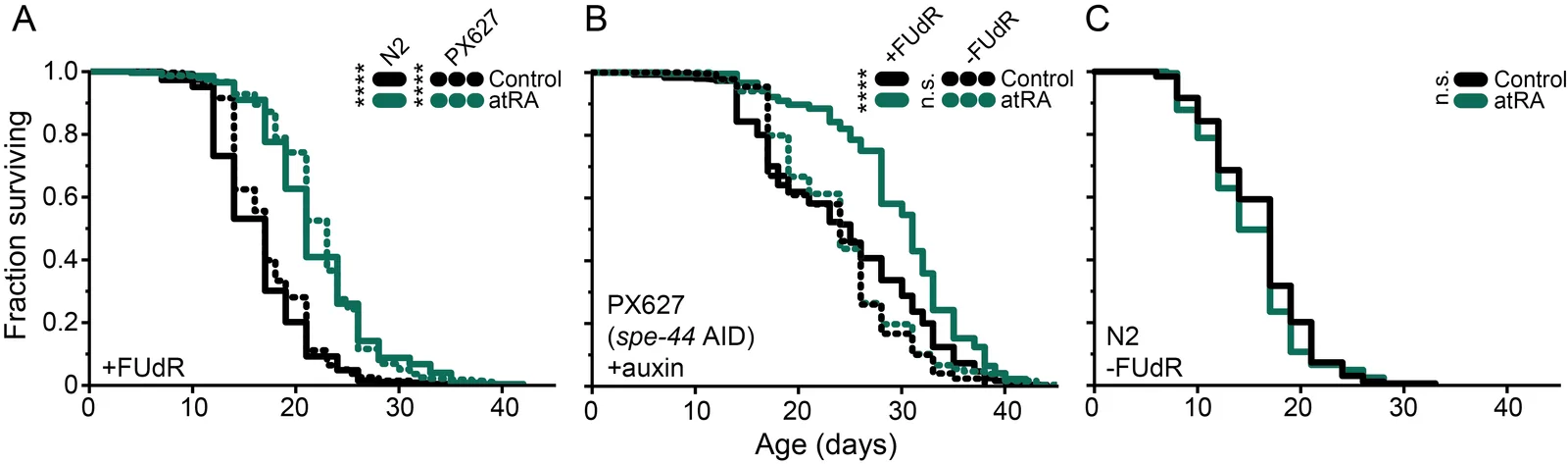



## Description

The *Caenorhabditis* Intervention Testing Program (CITP) is tasked by the NIA to characterize the effects of compounds on lifespan across a genetically diverse test-set of *Caenorhabditis* nematodes (Lucanic et al. 2017). The core premise of the CITP is that compounds which are efficacious across the panel (which includes *C. elegans* , *C. briggsae* , and *C. tropicalis* representatives) will be enriched for genetic background-independent modes of action.

All-trans retinoic acid (atRA) is a widely conserved signaling ligand involved in transcriptional regulation (Albalat and Cañestro 2009; Albalat 2009; Fonseca et al. 2020) via both retinoic acid receptor-dependent and -independent mechanisms. Evidence supports an endogenous atRA pathway in *C. elegans ,* although a clear retinoic acid receptor ortholog has not been described (Kostrouch et al. 1995; Garofalo et al. 2003; Chen et al. 2018). The CITP recently confirmed the longevity promoting impact of exogenously added atRA (Statzer et al. 2021; Banse et al. 2025) and documented that the conserved longevity factors AMPK/ *aak-2* , NRF2/ *skn-1* , AKT/ *akt-1 /2* , and HSF-1 / *hsf-1* act to confer atRA-dependent lifespan extension.

Given the positive effects we observed with atRA treatment, we sought to determine if atRA-mediated longevity effects were independent of the chemical sterilization regime commonly used in the *C. elegans* aging field. Because of the self-fertility of *C. elegans* , 5-Fluoro-2'-deoxyuridine (FUdR) is frequently used to prevent self-progeny from contaminating longevity assays (Hosono 1978; Mitchell et al. 1979). While FUdR simplifies scoring of age-synchronous adults in longevity studies, FUdR can shorten or lengthen lifespan depending on the dose and age at the time of exposure (Rooney et al. 2014; Wang et al. 2019). To address the potential impact of FUdR on atRA outcomes, we repeated our experiments using an alternative means of *C. elegans* sterilization—auxin-induced degradation of a degron-tagged *spe-44* in which the addition of auxin to the culture degrades SPE-44::degron to disrupt sperm maturation and confer sterility (Kasimatis et al. 2018). In the *spe-44::degron* strain treated with auxin, progeny is eliminated and FUdR can be omitted.

We first confirmed that atRA conferred lifespan effects in the *spe-44::degron* strain similar to wildtype under our standard experimental conditions, in which 51 µM FUdR was used to induce sterility ( Figure 1A ). We then tested auxin-sterilized animals grown in the absence of FUdR and observed that atRA did not extend lifespan when FUdR was omitted from the culture (median lifespan of 24 days for both control and treated animals, *p* =.21) ( Figure 1B ). The loss of efficacy of atRA is not due to the presence of auxin, however, since auxin-sterilized animals reared in the presence of FUdR exhibited a robust atRA lifespan extension (median lifespan of 25 days in control animals versus 31 days for treated animals, *p* <.0001) ( Figure 1B ). Notably, we did observe a lifespan increase with auxin itself (median lifespan of 25 days for the control on auxin versus 17 days without auxin, *p* <.0001), an effect that has been variably reported (Kasimatis et al. 2018; Loose et al. 2021). We returned to test *C. elegans* N2 lifespan in the absence of FUdR to show that the dependence on FUdR for atRA lifespan extension was not unique to the *spe-44::degron* genetic background ( *p* =.489) ( Figure 1C ). We conclude that inclusion of FUdR in the culture can have potentiating effects for atRA in *C. elegans* lifespan studies.

While we found that atRA induced robust longevity effects, with increased longevity observed in multiple *C. elegans* genetic backgrounds ( N2 , PX627 , MY16 , JU775 ) (Banse et al. 2025, this work), on different diets ( OP50-1 and HT115 ), and in manual and automated assays (Lifespan Machines), the cause of FUdR dependence is unclear. Our atRA findings constitute another example of known context-dependent cases of FUdR phenotypes. For example, FUdR can have significant lifespan-extending effects in *gas-1* (van Raamsdonk and Hekimi 2011), *cox-5B* (Lee et al. 2003; Suthammarak et al. 2009), *tub-1* ^21^ , and *exo-3* (Kato et al. 2017) genetic backgrounds. It is interesting that the first three of these loci intersect with mitochondrial biology: *gas-1* encodes a protein involved in the mitochondrial electron transport chain, *cox-5B* encodes an ETC complex V member, and *tub-1* mutants exhibit fat storage changes. Of note, previous characterization of PX627 auxin sterilization vs. FUdR for longevity studies identified differential effects on mitochondrial functions, with FUdR modulation of longevity specifically implicated in mitochondrial function (Dilberger et al. 2020). Metabolomics indicate that FUdR alters the *C. elegans* metabolic state (Davies et al. 2012), which might be anchored by altering the diet (McIntyre et al. 2021), direct effects on the animal (e.g., disruption of mitochondrial DNA replication), or both. EXO-3 is an apurinic/apyrimidinic exonuclease involved in DNA repair, which also could have a mitochondrial DNA connection. FUdR-induced DNA synthesis impairment does not profoundly impact biology in non-dividing adult somatic nuclei. However, FUdR can affect mitochondrial copy number—FUdR induces a concentration-dependent effect on mitochondrial DNA levels (but not on ATP levels, or mitochondrial morphology (Rooney et al. 2014)). As aging is known to change mitochondrial function/number/quality, FUdR may act as an enhancer of mitochondrial aging, which might sensitize *C. elegans* to anti-aging interventions that alter metabolism and mitochondrial function. It might be pointed out that mitohormesis-mediated longevity depends on *skn-1* and *hsf-1* (Schmeisser et al. 2013; Hwang et al. 2014), a transcription factor requirement that intersects with what we have documented for atRA lifespan extension (Banse et al. 2025). Future studies on the relationship between FUdR and compound efficacy across interventions may provide insight into the mechanistic role of FUdR in modulating lifespan intervention efficacy.

## Methods

We assayed lifespan in response to compound exposure in *C. elegans* N2 _PD1073 and the auxin tagged *spe-44::* degron strain PX627 (Kasimatis et al. 2018). In brief, animals were age-synchronized by timed egg-lays on standard 60 mm diameter Nematode Growth Media (NGM) plates and transferred at a density of 50 individuals per 35 mm treated plate in triplicate when they reached adulthood for two separate biological replicates. All-trans retinoic acid (atRA) was dissolved in DMSO and diluted appropriately such that addition of 132.5 µl of solution to 35 mm diameter plates containing NGM with lawns of *E. coli* OP50-1 (and either 0 µm or 51 µm FUdR) would generate the final compound concentration of 150 µM (0.25% DMSO final concentration). For assays where auxin was used to eliminate self-fertility, animals were maintained from hatching through day one of adulthood on assay plates with a final concentration of 1 mM auxin. For all experiments, animals were maintained at 20°C and moved to fresh plates on the first, second, and fifth day of adulthood, then once weekly afterward.

Statistical analyses were performed as previously described (Lucanic et al. 2017). In summary, survival was analyzed both with a generalized linear model using the lme4 package (v1.1-37), and a mixed-model Cox proportional hazards (CPH) approach using the coxme package (version 2.2-22; Therneau 2020) in the R statistical language (R Core Team 2021; v4.3.3). Compound effects were analyzed as a planned comparison between individuals exposed to compound (atRA) or vehicle control (DMSO) using the multcomp package (v1.4-30). All data and R scripts are available on figshare.com ( https://doi.org/10.6084/m9.figshare.28232681 ) aside from the N2 data in Figure 1 panel A which was previously published in Banse et al. 2025 ( https://doi.org/10.6084/m9.figshare.c.6320690 ).

## Reagents

Experiments were performed using *C. elegans* N2 _PD1073 (Banse et al. 2019; Yoshimura et al. 2019; Teterina et al. 2022) and PX627 ( *spe-44::degron* ; Kasimatis et al. 2018). For chemical interventions, atRA (Sigma-Aldrich PHR1187) was obtained in solid form and dissolved in DMSO (Sigma-Aldrich). Both FUdR (TCI America) and auxin (Alfa Aesar), when used, were added to the media during initial plate preparation.
